# The mental representation of true and false intentions: a comparison of schema-consistent and schema-inconsistent tasks

**DOI:** 10.1186/s41235-019-0173-4

**Published:** 2019-08-05

**Authors:** Sofia Calderon, Karl Ask, Erik Mac Giolla, Pär Anders Granhag

**Affiliations:** 1 Department of Psychology, University of Gothenburg, P.O. Box 500, 405 30 Gothenburg, Sweden; 2 Norwegian Police University College, Oslo, Norway

**Keywords:** Construal level theory, Action identification theory, True and false intentions, Mental representation

## Abstract

True and false intentions (i.e., lies and truths about one’s future actions) is a relatively new research topic, despite the high societal value of being able to predict future criminal behavior (e.g., in the case of an alleged terrorist attack). The current study examined how true and false intentions are mentally represented - the knowledge of which can aid the development of new deception detection methods. Participants (*N* = 151) were asked either to form a true intention about a future task (i.e., retrieve objects from an office) or to form a false intention about the same task (i.e., prepare a cover story about retrieving objects from an office) to conceal their actual intention (i.e., leave a secret note in the office). The schema consistency of the task was manipulated by presenting participants with a list of office supplies (schema-consistent) or random objects (schema-inconsistent) to be retrieved from the office. The abstractness of mental construal was operationalized as the number of categories used by participants to organize the task-relevant objects into thematic groups. We predicted, based on construal level theory (CLT) and action identification theory, that participants would mentally represent true intentions more concretely (i.e., use a larger number of categories) than false intentions, particularly for schema-inconsistent (versus schema-consistent) future tasks. The results of the study lend no support for these predictions. Instead, a Bayesian analysis revealed strong evidence in favor of the null hypothesis. The findings indicate that predictions from CLT do not readily translate into deception contexts. The results are discussed in light of recent failed attempts to apply CLT to research on true and false intentions, and highlight the need for alternative approaches to the topic.

**Electronic supplementary material:**

The online version of this article (10.1186/s41235-019-0173-4) contains supplementary material, which is available to authorized users.

## Significance

The ability to discriminate between true and false statements of intent is of utmost importance from a societal point of view. For example, in a police investigation, a correct veracity judgment of a statement of intention can prevent a planned future crime from happening. The field is new, and therefore lacks theoretical stringency. This is problematic since an understanding of the cognitive nature of intentions is key to developing methods for discriminating between truthful and deceptive statements. We tested if there are systematic ways in how true and false intentions are mentally represented. Informed by construal level theory (CLT) we reasoned that a true intention, which refers to a likely future action (since the person genuinely intends to perform the claimed action), should be relatively concretely represented. A false intention, on the other hand, which refers to an unlikely future action (since the person does not intend to carry out the claimed action), should be relatively abstractly represented. We expected a larger difference in mental abstraction when the intention referred to a schema-inconsistent task, than when the intention referred to a schema-consistent task. We found no support for our predictions. The aggregated findings from this and closely related studies suggest that true and false intentions instead may be represented at similar construal levels. If so, this calls for other innovative theoretical approaches to the important problem of how to unveil differences between true and false intentions.

It is of vast societal interest to distinguish between people’s true and false intentions -truths and lies about one’s future actions. Such knowledge can increase legal practitioners’ chances of preventing future crimes, for example by being able to spot markers of deception in a suspected terrorist’s statement (Granhag & Mac Giolla, 2014). Some thirty studies have to date been published on the topic of true and false intentions, and despite some promising findings (e.g., verbal markers of false intent; Mac Giolla, Granhag, & Liu-Jönsson, 2013), the field still lacks a stringent underlying theory. A recent attempt to rectify this was made by examining the underlying cognitive representations of true and false statements of intentions by applying predictions from CLT (Trope & Liberman, 2010) to the study of intentions (Calderon, Mac Giolla, Granhag, & Ask, 2017). This theoretical approach has the goal of improving verbal deception detection by revealing new cues to deceit. The current study builds on this novel work.

Calderon et al. (2017) proposed and tested the idea that that there may be systematic differences in the level of abstraction at which true and false intentions are mentally represented. They derived their prediction from the CLT framework (Trope & Liberman, 2010). With its roots in earlier theories on categorization (Rosch, Mervis, Gray, Johnson, & Boyes-Braem, 1976) and level of action identification (Vallacher & Wegner, 1987), CLT defines higher, abstract mental construals in terms of simpler scenes, and more inclusive categorization of objects and actions (i.e., fewer and broader categories). Lower, more concrete mental construals, on the other hand, involve more complex scenes, and less inclusive categorization of objects and actions (i.e., more and narrower categories; Trope & Liberman, 2010).

The specific contribution of CLT is that it explains under what circumstances people may adopt a more or less abstract mental representation of things. Specifically, it proposes that the further away a situation is from direct experience (i.e., the more psychologically distant), the more abstractly it will be mentally represented. For example, events happening further away in time (e.g., Liberman, Sagristano, & Trope, 2002) and space (e.g., Fujita, Henderson, Eng, Trope, & Liberman, 2006), and events happening to people that are percieved to be socially distant (e.g., Liviatan, Trope, & Liberman, 2008), have been shown to be represented more abstractly than events closer to here, now, and the self. In addition to the dimensions of temporal, spatial, and social distance, research has demonstrated how variations in subjective likelihood of events influence abstraction in a similar vein. Specifically, future events that are perceived as unlikely to occur have been found to be represented more abstractly than events perceived as likely to occur (Wakslak, Trope, Liberman, & Alony, 2006). There is an extensive number of empirical studies supporting the CLT assumptions. A meta-analysis including 310 effects of psychological distance on construal level from 125 experimental studies (Soderberg, Callahan, Kochersberger, Amit, & Ledgerwood, 2014) found a medium-sized effect (by the classic standards of Cohen, 1992) of psychological distance on how concretely or abstractly one represents an object or event. The effect was stable over cultures, settings, and types of distance (temporal, spatial, social, and likelihood distance).

Since a true intention typically is defined as a stated task that a person is committed to perform (Granhag, 2010), Calderon et al. (2017) argued that it naturally comes with a high perceived likelihood of occurring. Based on CLT, this means that a true intention should be represented relatively concretely. In contrast, since a false intention does not come with a commitment to perform the stated task, it comes with a low perceived likelihood of occurring. Therefore, relative to true intentions, false intentions should be represented more abstractly.

In two experiments, Calderon et al. (2017) introduced participants to simple tasks which they were told to either perform later (i.e., true intentions) or not to perform (i.e., false intentions). The participants were asked to provide statements saying that they were going to perform the tasks, thereby eliciting true and false statements. For example, tasks involved playing notes on a keyboard and attaching a poster to a wall. Established measures of construal level were used to assess the level at which true and false intentions were represented (e.g., participants’ preference for concrete or abstract alternative descriptions of the tasks). No differences in level of construal were found between true and false intentions. It was suggested that the null results were the effect of the tasks being too easy and schema-consistent for any differences to emerge in construal level. This explanation was derived from action identification theory^1^ (Vallacher & Wegner, 1987), which suggests that people tend to identify actions at a higher, more abstract, construal level in situations where actions are “non-disruptive” (e.g., easy, familiar, or schema-consistent). However, when an action is “disruptive” (e.g., a task which does not fit any existing mental script), people tend to turn to lower, more concrete, representations of the action. This is because concrete representations are more beneficial for action implementation in the latter case. In a study supporting this assumption, participants were asked to drink coffee from either normal-sized coffee cups or from unusually large and heavy cups (specifically designed to be disruptive of the action; Wegner, Vallacher, Macomber, Wood, & Arps, 1984). Wegner and colleagues found that participants drinking coffee from normal cups chose higher level, abstract, phrases to describe what they were doing (e.g., “becoming alert”, “satisfying my need”), whereas participants drinking coffee from the unusual cups favored lower-level, concrete descriptions (e.g., “putting a cup to my mouth”, “swallowing”).

Drawing on the findings of Wegner et al. (1984), Calderon et al. (2017) argued that both those stating true, and those stating false intentions, may hold relatively abstract mental representations when the tasks are non-disruptive. However, if a task is disruptive, truth tellers may adopt a more concrete construal level as it will be more functional for future action implementation. Those stating a false intention, on the other hand, who do not plan to perform their claimed intention, have little to gain by adopting a more concrete construal, even for schema-inconsistent tasks. To test this assumption, we asked the participants in the current study to prepare for a future task (i.e., true intention) or to plan a cover story to mask a secret mission (i.e., false intention). In order to examine the influence of the more theoretical concept of task disruptiveness, which comes in many guises (Vallacher & Wegner, 1987), we manipulated the specific case of schema consistency: The future task was either schema-consistent (i.e., collect office supplies from an office) or schema-inconsistent (i.e., collect random objects from an office). Construal level was measured as the number of thematic categories participants grouped the objects into, where a higher number of categories represents a more concrete construal (see, for example, Wakslak et al., 2006, “Exp. 1”, who used this dependent measure). We predicted that participants with a true intention would categorize these objects into more groups (indicative of a lower, more concrete level of construal) than those with a false intention, particularly for the schema-inconsistent task. Specifically, we predicted an interaction effect, such that the difference in construal level between true-intention and false-intention participants would be larger in the schema-inconsistent conditions than in the schema-consistent conditions.

## Method

### Participants

A priori power analysis indicated that 142 participants were needed to obtain statistical power of 80% to detect an interaction effect of Hedges’ *g* = 0.48 (α = 0.05). This effect size was based on the overall effect of psychological distance on mental construal level found in the most recent meta-analysis on the topic (Soderberg et al., 2014). To accommodate for attrition, 155 participants were recruited from a pool of voluntary participants at the psychology department of a large university in Sweden. They were invited to participate in a study in which they were going to perform an errand. Four participants were excluded - one due to language deficiencies, one who was uncomfortable with performing the task, and two who did not follow the instructions. This resulted in a final sample size of 151 participants, providing 84% power to detect an interaction effect of Hedges’ *g* = 0.48 (Soderberg et al., 2014) and 80% power to detect Hedges’ *g* = 0.46. Of the participants, 119 were women, 30 were men, and 2 did not specify their gender. Ages ranged from 18 to 72 (M = 27.56, SD = 9.92). As compensation, all participants received a gift card for a large shopping center to the value of 50 SEK (~ 6 USD) for their participation.

### Design and procedure

Participants were randomly assigned to one of four conditions in a 2 (intention: true versus false) × 2 (schema consistency: schema-consistent versus schema-inconsistent) between-groups design. Upon arrival at the laboratory, they were greeted by an experimenter who gave them an informed consent form to read and sign. They were then given written instructions for their errand. Tasks and measures described throughout the “Method” section are introduced in the order in which they occured in the experiment.

#### Instructions for the true intention group

Participants with a true intention were asked to imagine themselves working as a research assistant at the psychology department. They were instructed that they had been asked by their project leader to run an errand, which they were going to perform at the end of the study. The choice of asking participants to imagine being a research assistant was to create a framework for the task of gathering objects. Their errand was to go to an office located in the department building, gather a number of objects, put them in a bag, and hand them over to an employee. A list of all objects (27 items) was presented as a part of the instructions. In order to plan their errand they were also given a binder containing (1) a map of the building, (2) a photo of the interior of the office, (3) a keycard to access the part of the building where the office was situated, and (4) a note saying “I was here and fetched the items”, to be left in the office.

#### Instructions for the false intention group

Participants with a false intention were instructed that they were going to leave a secret note in an office located in the building. They were further informed that they might meet someone on their way to the office. Since the note was secret, they had to prepare a cover story to use in case they were asked what they were going to do in the office (i.e., to conceal their actual task). Participants were given a frame for this cover story (i.e., their false intention) which matched the true intention group’s actual task. That is, they were instructed that their cover story was to gather objects in a bag and hand the bag over to an employee, and were presented with the same list of objects (27 items) that was presented in the true intention condition. They were also given a binder containing (1) a map of the building, (2) a photo of the interior of the office, and (3) a keycard to access the part of the building where the office was situated. Finally, in contrast to the true intention group who received an innocuous note, participants in the false intention group were given an envelope with the word “secret” written on it, to be left in the office.

Participants in all conditions were told that they were allowed to bring the list of objects with them when they were going to perform the errand. After having read the instructions, the experimenter told them that they had 5 min to plan their errands, after which the dependent measure was collected.

#### Manipulation of schema consistency

To manipulate the schema consistency of the task, we created two different sets of objects: (A) a set of 27 office supplies and (B) a set of 27 random objects, not typically found in an office (see Table 1 for the sets of objects). This was done on the assumption that gathering office supplies from an office is relatively more schema-consistent than gathering random objects not typically found in an office. In order to tap into the broader concept task disruptiveness (Vallacher & Wegner, 1987), the lists of objects were also created with the aim that participants would find it easier to chunk the items in list A (versus list B) into categories, which in turn would make it easier (versus more difficult) to gather the objects. A pilot test (*N* = 15) supported these assumptions. In this test, participants were given both list A and B (the order of which was counterbalanced) and were asked to imagine that they were about to go to an office and gather some objects. There was a picture of the office next to the list, which was the same picture used for the actual experiment. Participants were asked to take a look at the list and to take a moment to think about which items belong together. They were then handed plastic notes and asked to place the objects that belong together next to each other. Participants had 2 min to complete the task, after which they rated how likely they were to find the objects at a typical office (1 = very unlikely to 7 = very likely), how easy/difficult it was to group the objects (1 = very easy to 7 = very difficult), how easy/difficult it would have been to gather the objects from the office (1 = very easy to 7 = very difficult), and how much time pressure they experienced during the task (1 = very little to 7 = very much). Participants found it more likely to find the objects in list A in an office (M = 5.93, SD = 1.03) than the objects in list B (M = 3.00, SD = 1.31), *t*(14) = 11.00, *p* < .001, *d* = 2.50, 95% CI 2.01, 3.00. They also found it less difficult to categorize the objects in list A into groups (M = 3.40, SD = 1.50) than the objects in list B (M = 4.40, SD = 1.18), although this difference failed to reach statistical significance *t*(14) = 2.01, *p* = .064, *d* = 0.75, 95% CI − 0.03, 1.53. In addition, participants estimated it to have been easier to gather the objects in list A (M = 3.53, SD = 1.55) than the objects in list B (M = 4.73, SD = 1.57), *t*(14) = 2.81, *p* = .014, *d* = 0.75, 95% CI 0.18, 1.35. As indirect support for differences in schema inconsistency, participants grouping list A (M = 3.33, SD = 1.54) experienced less time pressure than those grouping objects of list B (M = 4.20, SD = 1.47), *t*(14) = 2.16, *p* = .048, *d* = 0.58, 95% CI 0.01, 1.15. In addition, participants were asked to make relative judgments, comparing the two lists to each other: 13 out of 14 participants (93%) found it more likely to find the objects in list A in an office than the objects in list B, and 8 of 13 participants (62%) found it easier to group the objects in list A compared to the objects in list B, whereas one participant experienced no difference in difficulty grouping the objects.

**Table 1:** Objects used for the dependent measure

Schema-consistent task condition (A)	Schema-inconsistent task condition (B)
Sketchpad	Band aid
Flourescent pen	Almonds
Black notepad	Light bulb
Plastic pocket	Instant coffee
Stapler	Crispbread
Paper clip	Running shoes
Ball pen	Blanket
Crayon	Audiobook
Folder	Plate
Filing box	Orange
Thumbtack	Hand sanitizer
Tape	Cheetos
Yellow Post-it notes	Tooth brush
Thin fluorescent pen	Ping pong ball
Binder clip	Soap
Notepad	Deck of cards
Rubber	Lego
Pencil	Rubber duck
White A4 paper	Tea light
Scissors	Beanie
Pink fluorescent pen	Balloon
Pencil case	Scrabble
Large Post-it notes	Coffee spoon
Tipp-Ex	Fruit bowl
Clip	Glass bottle
Duct tape	Painting
Pin	Digital camera

#### Dependent measure

In order to assess construal level, participants were asked to categorize the objects in the list into groups. Object categorization tasks are a common way of measuring mental abstraction (Burgoon, Henderson, & Markman, 2013; Trope & Liberman, 2010), and have been used in numerous previous CLT studies (Fujita et al., 2006; Liberman et al., 2002; Steinhart, Mazursky, & Kamins, 2013; Wakslak et al., 2006; Wyer, Perfect, & Pahl, 2010). They rely on categorization theory, which assumes that people mentally categorize objects in a hierarchical manner into more or less inclusive categories (Rosch et al., 1976). For example, an owl can be categorized in the more inclusive category “animals” or the less inclusive category “birds”. The typical previous CLT study collected the dependent measure by handing participants a list of 38 objects (e.g., tent, matches, blanket) related to an imagined future task (e.g., a camping trip; Liberman et al., 2002, “Exp. 1”). Participants are asked to write down and circle the objects which they think belong together. A larger number of groups (i.e., less inclusive grouping) is indicative of a lower, more concrete mental representation. Other non-CLT studies have successfully used categorization tasks to assess level of abstraction. For example, Isen and Daubman (1984) manipulated participants’ affective state and handed them 14 color chips to categorize in as few or as many categories as they wanted. In line with the prediction they found that positive affect decreased the number of groups.

The current study used a combination of the two aforementioned approaches. Here, participants were handed 27 plastic cards, each with one of the 27 list objects written on it. Participants were then asked to categorize the objects into whatever groups seemed appropriate by placing them next to each other on the table in front of them. They had two minutes to complete this task. The written instructions for the grouping task were an amended version of instructions from previous studies using object grouping as a measure of construal level (Wakslak et al., 2006):

Your task is to group the objects by placing those objects that belong together next to each other. Place the plastic cards next to each other on the table in front of you. There is no “right” or “wrong” way of doing this. The number of groups is up to you to decide.

After the dependent measure had been collected, but before executing their plans, participants were intercepted and told that they would not actually perform the task. Instead, they were asked to answer a series of questions on a laptop computer. The experimenter specifically told participants in the false intention condition to answer truthfully on these questions, even though they previously had been instructed to lie.

#### Manipulation checks

As a manipulation check for the intention manipulation, participants were asked to what extent they believed they were actually going to go to the office (1 = not at all to 7 = to a very large extent), and to what extent they believed that they were actually going to gather the objects from the office (1 = not at all to 7 = to a very large extent). In addition, an oral manipulation check was collected before the dependent measure was introduced: All participants were asked “What are you going to do?”. Participants in the false intention conditions were additionally asked “Are you going to fetch the objects?”, to make sure that they understood that fetching the objects was merely a cover-story. As manipulation checks of schema consistency of the task, participants were asked how probable it is to find the objects in the list in a typical office (1 = very improbable to 7 = very probable) and how difficult it was to group the objects (1 = very easy to 7 = very difficult).

#### Control variables

Data were collected on a number of variables previously found to correlate with level of construal. Participants’ mood was measured using the Positive and Negative Affect Scale (PANAS; Watson, Clark, & Tellegen, 1988), which consists of self-report items on general mood and attentiveness. In addition, participants’ propensity for abstract thought was collected using the Behavioral Identification Form (BIF; Vallacher & Wegner, 1987). BIF lists a number of actions (e.g., “taking a test”) for which participants are asked to choose either an abstract alternative description (focused on why one performs the action; e.g., “showing one’s knowledge”) or a concrete alternative description (focused on how one performs the action; e.g., “answering questions”). Finally, a single item was included to measure the degree to which participants focused on memorizing the list of objects while they were performing the object grouping task (1 *=* to a very small extent to 7 = to a very large extent).

## Results

### Manipulation checks

Participants with false intentions (M = 6.45, SD = 1.10) and participants with true intentions (M = 6.35, SD = 1.45) believed to a similarly great extent that they were going to go to the office, *t*(149) = 0.48, *p* = .631, *d* = 0.08, 95% CI − 0.24, 0.40. As desired, participants with true intentions (M = 5.17, SD = 2.13) also believed to a greater extent than those with false intentions (M = 1.80, SD = 1.62) that they were going to collect the list of objects from the office *t*(149) = 10.95, *p* < .001, *d* = 1.78, 95% CI 1.40, 2.16. It should be noted that there was a trend indicating that, within the true-intention condition, participants in the schema-inconsistent condition (M = 4.78, SD = 2.25) believed to a lesser extent than those in the schema-consistent condition (M = 5.55, SD = 1.97) that they were going to gather the objects - an undesired mean difference indicating that the instruction to gather random objects made the task less believable. This test did, however, was not statistically significant, *t*(73) = 1.58, *p* = .119, *d* = 0.37 95% CI − 0.10, 0.83. In addition, all participants with true intentions told the experiment leader that they were going to the office to gather objects. All participants with false intentions told the experiment leader that that they were going to the office, but also that, in case they were to meet someone, they would deceptively claim that they would gather objects from the office. Taken together, the results indicate that the intention manipulation was successful.

Participants in the schema-consistent task condition (i.e., whose task concerned collecting or lying about collecting office supplies) rated it as more likely to find the objects in a typical office (M = 5.49, SD = 1.50), compared to participants in the schema-inconsistent task condition (i.e., whose task concerned collecting random objects from the office; M = 2.21, SD = 1.39), *t*(149) = 13.95, *p* < .001, *d* = 2.27, 95% CI 1.86, 2.68. This indicates that the manipulation was successful. Unexpectedly, however, there was no statistically significant difference between participants’ rated difficulty of grouping the office supplies (M = 2.84, SD = 1.58) and the rated difficulty of grouping the random objects (M = 3.21, SD = 1.35), *t*(149) = 1.55, *p* = .123, *d* = 0.25, 95% CI − 0.07, 0.57.

### Main analysis

Our main hypothesis was that participants with true (versus false) intentions would group task-relevant objects into a larger number of categories, and that this effect would be larger for the schema-inconsistent task than the schema-consistent task. A 2 (intention: true versus false) × 2 (schema consistency: schema-consistent versus schema-inconsistent) analysis of variance (ANOVA) was conducted on the number of groups in the object-grouping task to test this. Failing to support the prediction, there was no significant Intention × Schema consistency interaction effect, *F*(1, 147) = 1.37, *p* = .243, η^2^_p_ = .009, 90% CI .000, .051 (for means and standard deviations, see Table 2). Furthermore, there was no statistically significant main effect of the intention manipulation, *F*(1, 147) = 0.01, *p* = .944, η^2^_p_ < .001, 90% CI .000, .001, or a main effect of schema consistency, *F*(1, 147) < 0.01, *p* = .954, η^2^_p_ < 0.01, 90% CI .000, .001.

**Table 2:** Means (and SDs) of the dependent measure (number of object categories) by type of intention and schema consistency

	Schema consistency	
	Schema-consistent	Schema-inconsistent
True intention	6.79 (2.27)	6.27 (2.85)
False intention	6.32 (2.45)	6.79 (2.76)

Note, there were 27 objects in total. Number of categories ranged from 2 to 16. Fewer categories indicate a higher, more abstract construal

In order to estimate the support for the null hypothesis, Bayesian two-way ANOVA was conducted using the statistical software JASP (Love et al., 2015). Using Cauchy prior distribution (Wagenmakers et al., 2018), the analysis showed that the null model was preferred to a main effects and interaction model, with a Bayes factor of 80.7 in favor of the null. This means that the data provide strong evidence against the initial hypothesis that intention and schema consistency have an interactive effect (or any main effects) on level of construal, as operationalized with an object categorization task.^2^ The complete output from the Bayesian analysis is provided in Additional file 1.

### Control variables

A number of correlational analyses were conducted in order to investigate whether variables known to correlate with construal level were related to our outcome measure (number of object categories). Previous research suggests that a positive mood and propensity for more abstract thinking would be negatively correlated with the number of object categories, and that attentiveness and degree of memorization would be positively correlated with number of groups. Unexpectedly, there was no significant correlation between the number of object categories and self-reported positive mood (*r* = − .005. *p* = .949), propensity for abstract thinking (*r* = .069, *p* = .401), attentiveness (*r* = − .088, *p* = .280), or degree of memorization (*r* = − .126, *p* = .191).

## Discussion

Drawing on construal level theory (CLT; Trope & Liberman, 2010), action identification theory (Vallacher & Wegner, 1987), and suggestions from previous work within the field of intentions (Calderon et al., 2017), it was predicted that individuals with a true intention would represent aspects of the intention in more concrete terms than individuals with a false intention, and that this difference would be larger when the intention referred to a schema-inconsistent task as compared to a schema consistent task. This prediction was not supported: There were no differences in construal level between participants with true and false intentions, even when the future task was schema inconsistent. In addition, Bayesian analysis revealed strong evidence for the null hypothesis over the alternative hypothesis. Rather, the results are in line with those found by Calderon et al. (2017), who likewise found no support for a relationship between the veracity of intentions and their construal level. We suggest three competing explanations for the observed results, subsequently discussed in turn.

Our first explanation is that the results accurately indicate that true and false intentions are mentally represented at similar construal levels. There are recent preliminary findings supporting this scenario. Calderon et al. (2019) used an automated coding procedure and examined linguistic abstractness of intentions by conducting a mega-analysis of > 6000 statements of true and false intentions produced by over 500 individuals. Informed by CLT, they hypothesized that false intentions would be expressed more abstractly than true intentions, since variations in their mental abstraction should be mirrored in language use. Contrary to their predictions, they found no effect of veracity on verbal abstractness. Considering their large sample and relatively objective measure of concreteness, their finding supports the possibility that the current null result accurately shows that there are no differences in how abstractly true and false intentions are mentally represented. This explanation has important theoretical and practical implications. First, it means that the likelihood-as-psychological distance assumption does not seem to apply to the specific context of deception. Specifically, it indicates that a false (versus true) intention is not equal to a low (versus high) likelihood event, thereby limiting the scope of CLT. Second, it suggests that differences between statements of true and false intentions identified in previous studies (e.g., truth tellers’ statements being more detailed than liars’; Sooniste, Granhag, Knieps, & Vrij, 2013), may not be due to how abstractly future events are represented. Instead, it may be the result of other factors, such as differences in degree and quality of planning for the future intention (Mac Giolla, Granhag, & Liu-Jönsson, 2013).

A competing explanation for the observed results is that there are differences in mental construal between true and false intentions, but for various methodological reasons these were not uncovered. One such example is the dependent measure chosen for the current study. First, despite many previous examples of using the dependent measure (e.g., Isen & Daubman, 1984; Liberman et al., 2002), there was no correlation between the dependent measure and variables that theoretically should correlate with level of abstraction (i.e., mood, score on the behavioral identification form, degree of memorizing). This is surprising and indicates that the measure may not have successfully captured participants’ construal level. Second, a higher level of object categorization is only one of many predicted consequences of increased psychological distance (Soderberg et al., 2014). The association between psychological distance and construal level has been successfully uncovered using different perceptual measures (e.g., reaction times to visual stimuli; Bar-Anan, Liberman, & Trope, 2006), as well as more explicit behavioral measures (e.g., generation of arguments; Eyal, Liberman, Trope, & Walther, 2004). This means that, even though the current study failed to show a difference in breadth of object categorization, the mental representations of true and false intentions may still differ in other respects. In fact, Calderon et al. (2018) found that drawings of mental images accompanying false intentions were perceived as more abstract than drawings of mental images accompanying true intentions. This suggests that it may be important to use more indirect or holistic measures of abstractness in order to uncover differences in mental abstraction between true and false intentions. Future studies should test this possibility.

Another methodological concern is the manipulation of schema consistency. Action identification theory suggests that disruptive (e.g., schema-inconsistent) tasks should be identified at a lower, more concrete, construal level than non-disruptive tasks (Vallacher & Wegner, 1987). In the current study, participants in the schema-inconsistent condition should therefore have categorized the objects in a larger number of groups than those in the schema-consistent task condition, indicating a lower construal level. Unexpectedly, this was not the case. This suggests that the tasks were construed at similar levels across all groups. In addition, one manipulation check indicated that the manipulation was not successful (i.e., in creating differences in participants’ self-reported difficulty in grouping the objects). Taken together, this indicates that participants may not have experienced different levels of task disruptiveness. This is problematic, since an effect of veracity was only expected for disruptive tasks (i.e., the schema-inconsistent conditions). It should, however, be noted that our two manipulation checks typicality of the task and perceived difficulty grouping the objects did not correlate (*r* = − .033, *p* = .684). This indicates that they are two distinctly different concepts. Since the typicality measure, which was the manipulation check of primary interest, showed a large difference between groups we are not very concerned about the lack of difference in the second measure.

A final explanation for the current results is that the theoretical assumption behind the current study may lack validity. Even though there is extensive literature on CLT (Trope & Liberman, 2010), the specific assumption of likelihood as a form of psychological distance is understudied in relation to the other three dimensions (temporal, spatial, social). In the meta-analysis by Soderberg et al. (2014), only 8 out of 310 effects (0.03%) concerned the likelihood dimension. Nevertheless, it is, at least implicitly, given as much weight as the other dimensions within the theoretical framework. CLT is a vastly influential social-cognitive theory: introductory-level psychology books dedicate chapters to the theory (e.g., Kruglanski & Higgins, 2013), and the seminal review paper by Trope and Liberman (2010) has been cited on Google Scholar over 2887 times as of February 2019. Hence, if the likelihood assumption were unreliable, it would have large theoretical implications, as the term “psychological distance” would need to be revised. Furthermore, since the CLT framework is used to understand behaviors in more applied settings - such as organizational behavior (Wiesenfeld, Reyt, Brockner, & Trope, 2017), consumer choice (Liberman, Trope, & Wakslak, 2007), and pro-environmental behavior (McDonald, Chai, & Newell, 2015) - potential boundary conditions of CLT also have practical implications.

A recent failure to replicate a basic CLT finding - that low (versus high) perceived likelihood is associated with abstract (versus concrete) mental representations - support the possibility that it does not hold (Calderon, Mac Giolla, Ask, & Granhag: Subjective probability and the construal level of future events: a replication study of Wakslak, Trope, Liberman, & Alony (2006), submitted for publication). In brief, direct and high-powered replications of Wakslak et al.’s (2006) “Experiment 4 and 5” were unsuccessful: No effect of likelihood on construal level was observed. This indicates that the basic CLT assumption may be unreliable, or at least not as strongly supported by empirical evidence as previously thought. This, in turn, could mean that the theoretical rationale for predicting and testing an intention–construal-level link does not hold, as it would call into question the very idea of likelihood as a form of psychological distance. It is worth noting that both the failed replication and the previously mentioned failure to apply CLT to the deception contexts (Calderon et al., 2019) come from the same research laboratory. This raises the question as to whether some feature of the laboratory can explain these null findings. Considering the varying methods (replication study, mega-analysis), measures (object categorization, automated language coding), and data (both primary as well as secondary data from other research laboratories), we believe this to be unlikely. We do however encourage more attempts to replicate the effect of subjective likelihood of future events on construal level, in order to uncover potential boundary conditions of construal level theory.

A limitation with the current study worth noting is that the experimenter was not blind to the hypothesis and conditions during data collection. There is a risk that her expectations influenced the findings. The reason she was not blinded was (1) because we included an oral manipulation check, the phrasing of which was dependent on experimental conditions and (2) because she had been involved in planning the experiment. We believe it is unlikely, however, that the fact that she was not blinded had a major influence on the results: The interaction between the participant and the experimenter was minimal, and this interaction (i.e., instructions and questions) were directly taken from a written protocol. In addition, participants read most of the instructions.

In sum, we have proposed three competing explanations for the current null findings: (1) true and false intentions are mentally represented at similar construal levels; (2) there are differences in construal level between true and false intentions, but they were not uncovered due to methodological issues; and (3) the basic CLT assumption forming the basis of our prediction is invalid. Based on the arguments raised in our discussion, we believe that true and false intentions may not be represented at substantially different levels of mental abstraction. Future studies should, however, test the relative merit of the suggested explanations, as it remains an important issue to solve. Meanwhile, future research should continue to approach the topic of true and false intentions with innovative theoretical perspectives.

## Additional file

Additional file 1: **Table S1.** Means and (standard deviations) over the four conditions in a sample restricted to participants who did not fail the manipulation check of believability of performing the task (*N* = 117). **Table S2.** Bayesian ANOVA, Model Comparison for Dependent Variable (Number of Object Groups) for the complete sample (*N* = 151). **Table S3.** Bayesian ANOVA, Analysis of Effects for Dependent Variable (Number of Object Groups) for the complete sample (*N* = 151). (DOCX 21 kb)

## Data Availability

Data and materials are available on https://osf.io/ch3d4/.

## Footnotes

1 Action identification theory is an influential social-cognitive theory. As of February 2019, the review article of Vallacher and Wegner (1987) had 2008 citations of Google Scholar, and social psychology books dedicate entire chapters to it (e.g., Kruglanski & Higgins, 2013).

2 We conducted two additional analyses (frequentist analysis and Bayesian two-way ANOVA) for which we excluded participants who “failed” the manipulation check of participants’ believability of gathering the objects. That meant excluding participants in the true intention condition with values ≤ 5 on the manipulation check, and participants in the false intention condition with values ≥ 3 (on a 7-point scale). The new smaller sample revealed a similar trend of the data (i.e., non-significant interaction and a Bayes factor in favor of the null; see Additional file 1 for the entirety of these analyses).
