# Supplementary material for: The mental representation of true and false intentions: a comparison of schema-consistent and schema-inconsistent tasks
Source: Cogn Res Princ Implic. 2019 Aug 5;4:29. doi: 10.1186/s41235-019-0173-4 (PMC6682835; doi:10.1186/s41235-019-0173-4)
Supplement: Supplementary file 1 — Table S1. Means and (standard deviations) over the four conditions in a sample restricted to participants who did not fail the manipulation check of believability of performing the task (N = 117). Table S2. Bayesian ANOVA, Model Comparison for Dependent Variable (Number of Object Groups) for the complete sample (N = 151). Table S3. Bayesian ANOVA, Analysis of Effects for Dependent Variable (Number of Object Groups) for the complete sample (N = 151). (DOCX 21 kb) [file 41235_2019_173_MOESM1_ESM.docx]

**Additional file 1**

We conducted an additional analysis for which we excluded participants who “failed” the following manipulation check question: “To what extent did you believe you were going to go to the office and gather the objects?”. Specifically, we only included participants in the true intention condition with values ≥ 5 on the manipulation check, and participants in the false intention condition with values ≤ 3 (on a 7-point Likert type scale). The new sample was 117 (*n*_disruptive-_*_t_*_rue_= 23, n_disruptive-false_= 33, *n*_non-disruptive-_*_t_*_rue_= 29, *n*_non-disruptive-false_= 32). The below results should be viewed in light of lack of power (i.e., 72% power to detect the targeted interaction effect).

A 2 (intention: true vs. false) × 2 (task disruptiveness: non-disruptive vs. disruptive) Analysis of Variance (ANOVA) was conducted in order to test the hypothesis that participants with true (vs. false) intentions would group task-relevant objects into a higher number of categories, particularly those in the disruptive-task condition. Failing to support this prediction, there was no significant Intention × Task Disruptiveness interaction effect, *F*(1, 116) = 0.89, *p* = .346, η^2^_p_ = .008, 90% CI [.000, .005] (for means and standard deviations, see Table 1). Furthermore, there was no statistically significant main effect of the intention manipulation, *F*(1, 116) = 0.01, *p* = .905, η^2^_p_ < .001, 90% CI [.000, .025], or a main effect of task disruptiveness, *F*(1, 116) = 0.07, *p* = .788, η^2^_p_ = 0.01, 90% CI [.000, .005].

To estimate the evidence for the null hypothesis, we conducted a Bayesian Analysis of Variance (ANOVA). Based on a Cauchy prior distribution, the analysis revealed a Bayes Factor of 70.1 in favor of the null. This means that the data provide strong evidence against the initial hypothesis that intention and task disruptiveness have an interactive effect (or any main effects) on level of construal (see Table 1 and Table 2 for the complete output from the analyses).

Table S1. Means and (standard deviations) over the four conditions in a sample restricted to participants who did not fail the manipulation check of believability of performing the task (*N* = 117)

|  | Task disruptiveness | |
| --- | --- | --- |
|  | Non-disruptive | Disruptive |
| True intention | 6.90 (2.37) | 6.57 (3.20) |
| False intention | 6.38 (2.04) | 7.00 (2.88) |

| Table S2. Bayesian ANOVA, Model Comparison for Dependent Variable (Number of Object Groups) for the complete sample (*N* = 151) | | | | | | | | | | | | | | | | |
| --- | --- | --- | --- | --- | --- | --- | --- | --- | --- | --- | --- | --- | --- | --- | --- | --- |
| **Models** | | | | | | | **P(M)** | | **P(M\|data)** | | **BF _M_** | | **BF _01_** | | **error %** | |
| Null model | | | | | |  | 0.200 |  | 0.718 |  | 10.160 |  | 1.000 |  |  |  |
| Intention | | | | | |  | 0.200 |  | 0.126 |  | 0.577 |  | 5.693 |  | 5.376e -6 |  |
| Schema Consistency | | | | | |  | 0.200 |  | 0.126 |  | 0.576 |  | 5.703 |  | 5.385e -6 |  |
| Intention + Schema Consistency | | | | | |  | 0.200 |  | 0.022 |  | 0.089 |  | 33.004 |  | 1.126 |  |
| Intention + Schema Consistency + Intention   ✻   Schema Consistency | | | | | |  | 0.200 |  | 0.009 |  | 0.036 |  | 80.654 |  | 2.660 |  |
|  | | | | | | | | | | | | | | | | |
| Table S3. Bayesian ANOVA, Analysis of Effects for Dependent Variable (Number of Object Groups) for the complete sample (*N* = 151) | | | | | | | | | |  |  |  |  |  |  |  |
| **Effects** | | **P(incl)** | | **P(incl\|data)** | | **BF _Inclusion_** | | | |  |  |  |  |  |  |  |
| Intention |  | 0.600 |  | 0.157 |  | 0.124 | | |  |  |  |  |  |  |  |  |
| Schema Consistency |  | 0.600 |  | 0.156 |  | 0.124 | | |  |  |  |  |  |  |  |  |
| Intention   ✻   Schema Consistency |  | 0.200 |  | 0.009 |  | 0.036 | | |  |  |  |  |  |  |  |  |
|  | | | | | | | | | |  |  |  |  |  |  |  |
